# Clarifying mammalian RISC assembly *in vitro*

**DOI:** 10.1186/1471-2199-12-19

**Published:** 2011-04-29

**Authors:** Grace S Tan, Barry G Garchow, Xuhang Liu, David Metzler, Marianthi Kiriakidou

**Affiliations:** 1 Department of Medicine, Division of Rheumatology, University of Pennsylvania School of Medicine, Philadelphia, PA, USA; 2 Department of Pathology, University of Pennsylvania School of Medicine, Philadelphia, PA, USA

## Abstract

**Background:**

Argonaute, the core component of the RNA induced silencing complex (RISC), binds to mature miRNAs and regulates gene expression at transcriptional or post-transcriptional level. We recently reported that Argonaute 2 (Ago2) also assembles into complexes with miRNA precursors (pre-miRNAs). These Ago2:pre-miRNA complexes are catalytically active *in vitro *and constitute non-canonical RISCs.

**Results:**

The use of pre-miRNAs as guides by Ago2 bypasses Dicer activity and complicates *in vitro *RISC reconstitution. In this work, we characterized Ago2:pre-miRNA complexes and identified RNAs that are targeted by miRNAs but not their corresponding pre-miRNAs. Using these target RNAs we were able to recapitulate *in vitro *pre-miRNA processing and canonical RISC loading, and define the minimal factors required for these processes.

**Conclusions:**

Our results indicate that Ago2 and Dicer are sufficient for processing and loading of miRNAs into RISC. Furthermore, our studies suggest that Ago2 binds primarily to the 5'- and alternatively, to the 3'-end of select pre-miRNAs.

## Background

MicroRNAs (miRNAs) are small (~22 nucleotide) noncoding RNAs that associate with Argonaute proteins in ribonucleoprotein complexes (miRNPs or RISCs) [1-5]. MicroRNAs play diverse regulatory roles in development and physiological cellular functions [6-8]. In addition, miRNAs are involved in a wide spectrum of human diseases including cancer, cardiovascular and autoimmune inflammatory conditions [8-14].

Post-transcriptional nuclear processing of primary miRNA transcripts (pri-miRNAs) by the RNAse III enzyme Drosha and its RNA-binding partner, DiGeorge syndrome Critical Region gene 8 protein (DGCR8) [15-18], generates ~ 65-75 nucleotide (nt) hairpin-structured miRNA precursors (pre-miRNAs). Pre-miRNAs are subsequently bound to the RAN-GTP protein Exportin-5 and are transported to the cytoplasm [19-22] where they undergo processing by the RNAse III enzyme Dicer into ~ 22 nt RNA duplexes [23,24]. TAR RNA binding protein (TRBP) facilitates Dicer processing of pre-miRNAs [25-28]. Ago proteins act as RNA chaperones facilitating unwinding of RNA duplexes and loading of single-stranded miRNAs into Ago complexes [29,30]. The mechanisms involved in miRNA strand selection in mammals are poorly understood.

We previously reported that endogenous Ago2-pre-miRNA (pre-miRNPs) complexes are detected in cytoplasmic and nuclear extracts of human cells, and that pre-miRNPs are enriched in *Dicer-null *cells [31]. We showed that recombinant pre-miRNPs function as RISCs *in vitro*. This finding complicated our *in vitro *RISC reconstitution studies since recombinant Ago2 in complex with pre-miRNAs cleaves target RNAs bypassing the requirement for Dicer activity. In this study, we identified RNA targets that are not cleaved by Ago2:pre-miRNA complexes. We demonstrated that Dicer and Ago2 are sufficient for *in vitro *recapitulation of the cytoplasmic miRNP assembly and that TRBP is dispensable. We also showed that select Ago2:pre-miRNA complexes are active against 5'-as well as 3'-arm RNA targets, suggesting that Ago2 binds primarily to the 5'- and alternatively, to the 3'-end of select pre-miRNAs.

## Results

### Recombinant Ago2:pre-miR-24-1 complex directs cleavage of a 5'- but not of a 3'-arm target

We previously reported that mammalian Ago2 directly binds to pre-miRNAs and forms active, non-canonical, RISC (pre-miRNPs) *in vitro *[31]. Ago2 can only use the hairpin-like pre-miRNAs as guides for target cleavage when spontaneous or Ago2-mediated dissociation of the hairpin arms permits target annealing to pre-miRNAs. Interestingly, two recent studies demonstrated RNA chaperone activity of human Ago proteins against RNA duplexes [29,30].

Although the function and biological significance of endogenous pre-miRNPs is unclear, non-canonical RISC activity of pre-miRNPs complicates studies of RISC reconstitution. To bypass this alternative *in vitro *pathway, we first asked whether non-canonical RISC is also active against targets complementary to the 3'-arm of pre-miRNAs.

**Figure 1:**
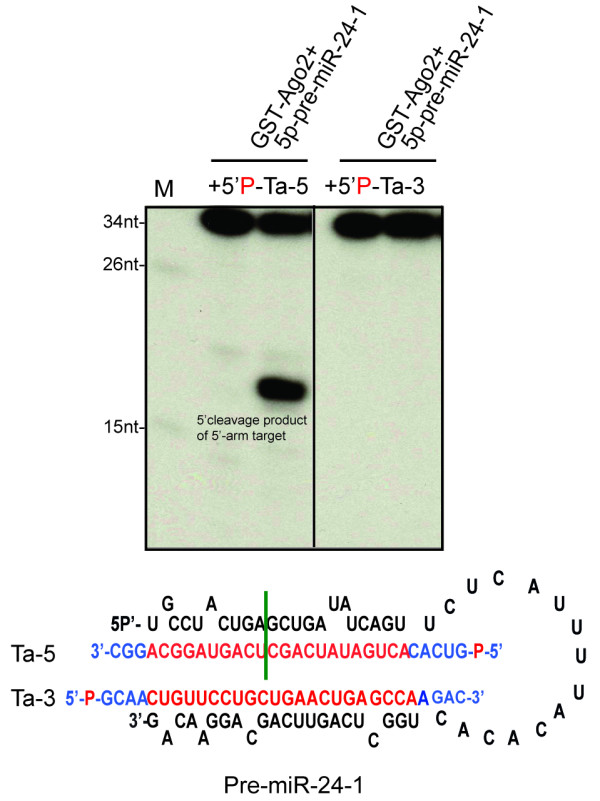
**Recombinant Ago2:5'P -pre-miR-24-1 complex is active against a 5'- but not a 3'-arm target**. Cleavage assay results of recombinant Ago2 pre-incubated with 5'-P-pre-miR-24-1 before the addition of radiolabeled 5'-arm (Ta-5) or 3'-arm (Ta-3) target. Predicted sizes of 5'-end cleavage products of 5'- and 3'-arm targets are 17nt and 14nt respectively. Radiolabeled pBR322/MspI (M) was used as size marker.

To test our hypothesis, we preloaded purified GST-Ago2 with pre-miR-24-1 in near stoichiometric amounts and added a 5'-P^32^-radiolabeled target complementary to either the 5'- or the 3'-arm of pre-miR-24-1. As expected, efficient 5'-arm target cleavage was detected in the absence of Dicer, whereas 3'-arm target cleavage was not observed (Figure 1). This suggests preferential activity of pre-miR-24-1:Ago2 complex against targets complementary to the proximal end of pre-miR-24-1.

### Ago2 and Dicer are sufficient for *in vitro *recapitulation of cytoplasmic steps of miRNA biogenesis

**Figure 2:**
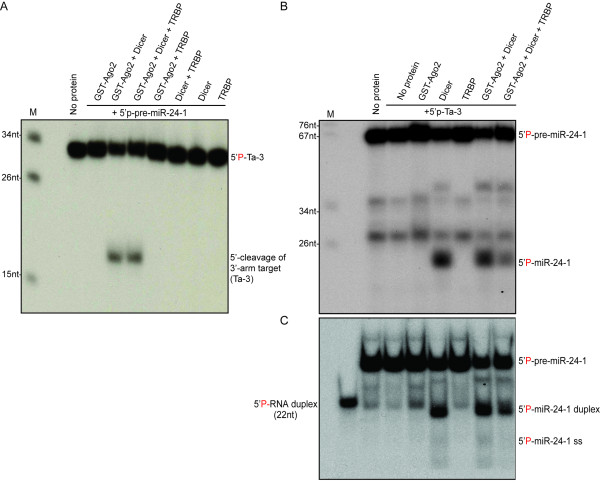
Recombinant Ago2 and Dicer are sufficient to recapitulate processing of 5'P -pre-miR- 24-1 and loading of miR-24-1* strand into Ago2 *in vitro*. **A**. Results of cleavage assays using recombinant Ago2, Dicer, and TRBP (in combinations as indicated) pre-incubated with 5' -P-pre-miR-24-1, prior to the addition of radiolabeled 3' -arm target (Ta-3). **B**. Experiments were performed as in (A), except with radiolabeled pre-miR-24-1 and unlabeled 3' -arm target (Ta-3). 50% of the total volume of each reaction was analyzed on 15% Urea PAGE and RNAs were detected by autoradiography. **C**. The remaining 50% of each reaction was analyzed by native RNA gel electrophoresis. Pre-annealed 22nt RNA duplex was used as size marker.

Although *in vitro *reconstitution of mammalian RISC with recombinant Ago2, Dicer and TRBP has been previously reported, it is unclear whether in this study RISC was formed by Ago loaded with a Dicer-generated miRNA or unprocessed pre-miRNA [32]. Having demonstrated inactivity of Ago2-pre-miR-24-1 against a 3'-arm target, we were in a position to test RISC loading, by circumventing the non-canonical pathway. Ago2 was incubated with 5'-P-pre-miR-24-1 and Dicer, but not TRBP, then provided with a 5'-radiolabeled 3'-arm RNA target (Ta-3). Reaction products were analyzed by Urea-PAGE. Efficient cleavage of the radiolabeled Ta-3 was observed (Figure 2A), indicating TRBP-independent loading of the 3'-arm product of pre-miR-24-1 into Ago2. Inclusion of TRBP to the reconstitution reactions did not affect RISC activity (Figure 2A). These results indicate that Ago2 and Dicer alone were sufficient for canonical RISC loading *in vitro*.

In order to demonstrate that the 5'-arm product of pre-miR-24-1 was also loaded into Ago2, in addition to the 3'-arm product, the same reactions were repeated using 5**'**-radiolabeled pre-miR-24-1 and unlabeled RNA target. Labeled RNAs were analyzed by denaturing and native PAGE (Figure 2B and 2C). As expected, Dicer, but not Ago2 or TRBP, processed pre-miR-24-1 to ~21 nucleotide miR-24-1:miR-24-1* duplex, which was then dissociated by Ago2 and formed canonical RISC loaded with either miR-24-1 or miR-24-1* (Figure 2C and 2A). Although when these reactions were repeated with labeled pre-miR-24-1 and unlabeled target RNA, TRBP appeared to have a mild inhibitory effect on Dicer (Figure 2B), such effect was not observed when pre-miR-30 was used instead of pre-miR-24-1 (Additional File 1) or when RISC activity was tested (Figure 2A). Irrespective of regulatory activity, the presence of TRBP was also not essential for pre-miR-30a RISC loading reconstitution (Additional File 1). Together, our results indicate that recombinant Ago2 and Dicer are sufficient to recapitulate RISC assembly *in vitro*. We demonstrated unwinding of Dicer-generated miRNA duplexes in the presence of Ago2 and RISC loading with single-stranded miRNAs deriving either from the 3'-arm (as indicated by RISC activity shown in Figure 2A and Additional File 1A), or the 5'- arm of pre-miR-24-1 (as shown in Figure 2B and 2C and Additional File 1B and 1C). TRBP is not essential for pre-miRNA processing by Dicer, unwinding of the RNA duplex or loading of miRNA or miRNA* into Ago2.

### RISC activity of select pre-miRNPs is guided against 5'-and 3'-arm targets

**Figure 3:**
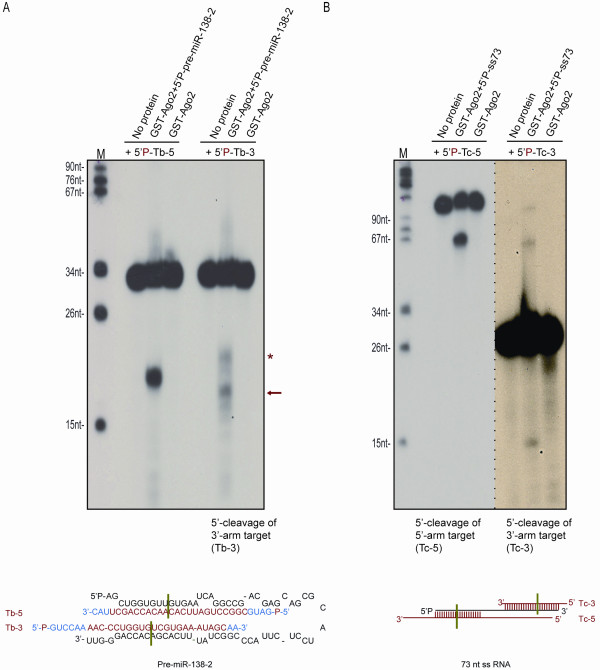
**Recombinant Ago2: pre-miR-138-2 and Ago2:73nt ssRNA complexes direct 5' - and 3' -arm target cleavage**. **A**. Results of cleavage assay with recombinant Ago2 pre-incubated with gel-extracted 5' -P-pre-miR-138-2, before the addition of radiolabeled gel-purified RNA targets complementary to its 5' - (Tb-5) or 3' -arm (Tb-3). Predicted size of 5' - cleavage product of Tb-5 and Tb-3 is 17 nt and 16 nt respectively. **B**. Experiments were performed as in (A), except that a gel-purified 73nt single-stranded (ss) RNA was pre-incubated with Ago2 before the addition of radiolabeled 5' -end (Tc-5) or 3' -end target (Tc-3). Predicted sizes of 5' - end cleavage products of 5' -arm and 3' -arm targets are 60 nt and 15 nt respectively.

We tested several pre-miRNAs to demonstrate that a non-canonical Dicer-free Ago pre-miRNP is preferentially active against a 5'-arm target. Surprisingly, we observed 3'-arm target cleavage by select pre-miRNA-guided RISCs. Pre-miR-24-1 and pre-miR-30a consistently demonstrated 5'- but not 3'-arm target cleavage, while pre-miR-138-2 and pre-miR-103-2 guided cleavage of targets complementary to both arms. In the case of pre-miR-138-2, the 17nt 5'- cleavage product of target Tb-5 (complementary to the 5'-arm of pre-miR-138-2), corresponds, as expected, to cleavage across the nucleotide in position 10 from the 5'-end of pre-miR-138-2 (Figure 3A). The 5'- product of the 3'-arm target Tb-3 (16 nt) corresponds to cleavage across the nucleotide in position 10 from the 3'-end of pre-miR-138-2 (Figure 3A). An additional 18nt 5'-product is seen, indicating cleavage across position 12 from the 3'-end. These findings suggest that, *in vitro*, in addition to binding to the 5'-phosphorylated end, recombinant Ago2 likely binds to the 3'-unphosphorylated end of pre-miR-138-2. Imprecise cleavage of the 3'-arm target resulting in two different size products suggests weak Ago2 binding to the unphosphorylated base of the 3'-end. An alternative explanation of these findings would be that Ago2 processes pre-miRNAs and is then loaded with guide RNAs deriving from their 3'- arm. However, we have previously [31] and currently demonstrated that such processing is not detected *in vitro *(Figure 2B and Additional File 1B) at least with the conditions and pre-miRNAs we tested using purified, Dicer-free, recombinant Ago2, ruling out this alternative scenario.

Pre-miR-103-2, like pre-miR-138-2, directed cleavage of targets complementary to its 5'- and 3'- arms (Additional File 2). Imprecise cleavage of the 3'-arm target was noted again, as with pre-miR-138-2. In pre-miR-103-2-guided cleavage, addition of recombinant TRBP was not crucial for the use of the 5'- or 3'- pre-miRNA arm by Ago2 (Additional File 2).

When Ago2 was loaded with a 73-nucleotide single-stranded, unstructured RNA, both 5'- and 3'- end targets were cleaved, although 3'-end target cleavage was significantly less efficient (Figure 3B, and Additional File 3 for guide:target RNA alignment). This again suggests that Ago2 binds primarily, as expected, to the 5'-phosphorylated base of the guide RNA and can also bind to a small extent to the 3'- unphosphorylated base of the single-stranded guide RNA.

**Figure 4:**
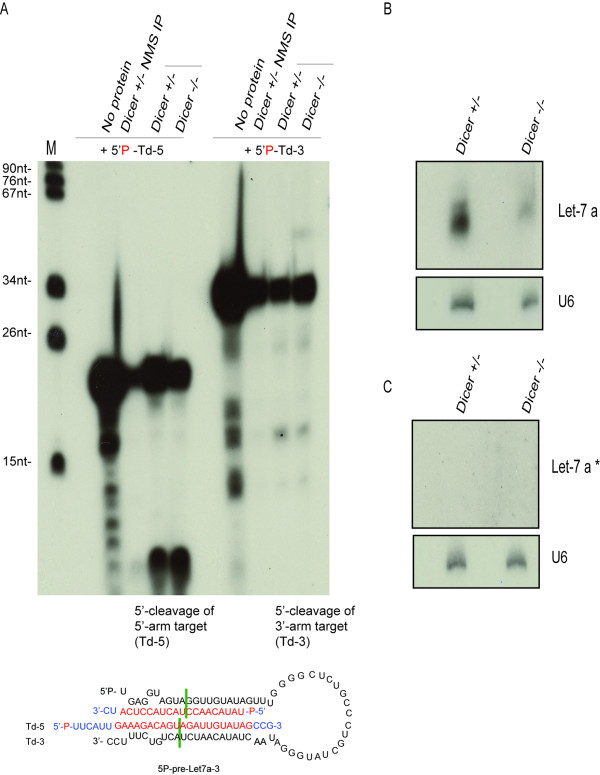
**Endogenous pre-Let7a-3:Ago2 complexes guide 5'- and 3'- arm target cleavage *in vitro***. **A**. Endogenous pre-miRNPs were immunopurified using anti-Ago (2A8) antibody or from *Dicer^+/- ^or Dicer^-/- ^*mouse embryonic fibroblasts (MEFs). Non-immune mouse serum (NMS) was used as control. 30% of the total volume of agarose G beads from each immunoprecipitation (IP) was then incubated either with radiolabeled 5' -arm (Td-5) or with 3' -arm target (Td-3) and then treated with proteinase K. Predicted size of 5 -cleavage product of 5' -arm and 3' -arm target is 9 nt and 16 nt respectively. **B **and **C**. Expression of let-7a or let-7a * was detected by Northern blot of total RNA (20 μg/lane) isolated from *Dicer^+/- ^or Dicer^-/- ^*MEFs, using DNA antisense probes. For loading control, let-7a and let-7a * expression was normalized against the expression of U6 RNA.

Endogenous Ago2-pre-miRNA complexes are detected in nuclear and cytoplasmic extracts of mammalian cells [31]. We have previously shown that in *Dicer-null *mouse embryonic fibroblasts (MEFs), endogenous Agos are loaded with unprocessed pre-miRNAs [31]. To test whether Ago2 binds to the 3'- end of endogenous pre-miRNAs, we immunoprecipitated Ago complexes from *Dicer^-/- ^*MEFs using the 2A8 antibody[33] and performed *in vitro *RISC assays. In this inducible cell line a small amount of let-7a is detected (Figure 4B and[31]) even when Dicer is undetectable (Additional File 4). However, let-7a*, which normally originates from the 3'-arm of pre-let-7a, is not detected in total RNA from *Dicer *^+/- ^or *Dicer^-/- ^*MEFs (Figure 4C). To test the RISC activity of endogenous pre-let7-a RNPs, we provided RNA targets complementary to the 5'- and 3'-arm of pre-let-7a. In addition to the 5'- arm cleavage, a 3'-arm target cleavage product was observed, likely generated by RISC activity of pre-let-7a RNPs, rather than by canonical, let-7a* miRNPs (Figure 4A), suggesting that endogenous pre-let-7a associates to Ago2 via either its 5'-phosphorylated or its 3'- unphosphorylated end.

## Discussion

Most endogenous pre-miRNAs are normally processed by Dicer and consequently functional miRNPs are more abundant than pre-miRNPs [31]. We have previously shown that canonical pre-miRNAs associate with Ago2 and that *in vitro*, Dicer-free Ago2 does not process pre-miRNAs into mature miRNAs or any intermediate products [31]. It was subsequently shown that Ago2 binds to pre-miR-451 and mediates its processing via a non-canonical, Dicer-independent pathway [34,35]. Unlike most mammalian miRNAs, pre-miR-451 has a unique stem structure, with a perfect sense:antisense complementarity, resembling a 17nt siRNA duplex. Our present and previous studies [31] indicate that pre-miRNAs with stem bulges are not processed by Ago2, but are used as guides for RISC activity *in vitro*. Our unexpected findings, also reported by Yoda et al [30], complicate *in vitro *dissection of RISC biogenesis and function.

In this study, we identified pre-miRNPs inactive against 3'-arm targets and this finding allowed us to demonstrate that Dicer and Ago2 are sufficient to process pre-miRNAs, unwind miRNA duplexes and load single-stranded miRNAs into Ago2. TRBP was dispensable for all three steps *in vitro*, arguing against an essential role for this protein in RISC reconstitution or miRNA strand selection but not excluding a regulatory function. Several studies have shown regulation of miRNA biogenesis by auxiliary factors (Reviewed in [36]). It has been previously shown that TRBP regulates pre-miRNA processing by Dicer [25-28]; furthermore, it has been proposed that TRBP is necessary to recruit Ago2 to the Dicer/substrate complexes [37]. Earlier reports suggested that TRBP in complex with Ago and Dicer is required for RISC loading reconstitution *in vitro *[32]. Our studies indicate that TRBP is not essential for this process.

Apart from clarifying the minimal factors required for mammalian RISC loading, our results suggest that pre-miRNAs bind to Ago2 via either their 5'- or 3'- end. Crystal structure studies have shown that the 3'- [38,39] and the 5'- end [40] of miRNAs bind to Argonaute proteins and that there is a strong preference for U or A as a first 5'- end base [41,42]. It is likely that pre-miRNAs are anchored within Ago2 in a similar fashion. We tested four differently structured pre-miRNAs and the size of the 5'-arm target product consistently corresponded to the expected cleavage across the nucleotide in position 10 from the 5'-end of the pre-miRNA. Most of the 3'-arm target products corresponded also to cleavage across the 10^th ^nucleotide from the 3'-end. However, additional products were detected suggesting imprecise cleavage, likely due to weak Ago2 binding to the 3'-unphosphorylated pre-miRNA end. It is unlikely that these RNA products represent degradation artifacts, as all the radiolabeled RNA oligos were gel-purified.

## Conclusions

Our results indicate that Ago2 and Dicer are sufficient for processing and loading of miRNAs into RISC. Furthermore, our studies suggest that Ago2 binds primarily to the 5'-and alternatively, to the 3'-end of select pre-miRNAs. Results from our experiments using immunopurified pre-miRNPs from *Dicer-null *cells also support this alternative mode of endogenous pre-miRNA binding to Ago2. Endogenous pre-miRNPs accumulate in Dicer-deficient cells, likely because the secondary structure of pre-miRNAs and/or their binding by Ago2 protects them from degradation; normally, Ago2 containing pre-miRNPs are not abundant [31]. Since it is unlikely that miRNPs and pre-miRNPs exert redundant regulatory functions on gene expression, our *in vitro *findings should be cautiously interpreted, until the function of pre-miRNPs and biological significance of their RISC activity are elucidated.

## Methods

### Expression and Purification of GST-Ago2 and GST-TRBP

Recombinant GST-Ago2 and GST-tagged human TRBP2 isoform a (NM_134323.1) were expressed using the baculovirus protein expression system and purified as described previously [31]. For protein purification, the baculovirus-infected *Sf9 *cell pellets were sonicated and recombinant protein was purified by Glutathione sepharose beads, washed and further purified by anion exchange chromatography. Silver nitrate gel analyses and pre-miRNA processing assays were performed with every protein batch to ensure purity of recombinant Ago2 or TRBP and absence of Dicer (or Ago2 from TRBP preparations) or other associating proteins. Western Blot analyses with primary antibodies against Dicer (Novus NBP1-06520, 1:2000), 2A8 ([33], 1:1000) and TRBP ([25], 1:1000) were also performed (Additional File 5).

### 5'- labeling of RNAs

RNA oligonucleotides were 5'-end radiolabeled with T4 polynucleotide kinase (NEB M0201S) as previously described [43,44].

### Cleavage and Reconstitution Assays

Cleavage assays were performed as previously described [31]. For reconstitution assays, 20 ng recombinant GST-Ago2, GST-TRBP and/or Dicer (Ambion AM2212) were pre-incubated with 20 nM of 5'-P-pre-miRNA at 37°C for 30 min. ~5 nmol of gel extracted, 5'- radiolabeled RNA target was then added to the reactions, which were then incubated at 37°C for additional 60 min. Cleavage products were then analyzed on 15% UREA PAGE. A 5' radiolabeled size marker (pBR322/MspI, NEB) was used for size control and radiolabeled cleavage products were detected by autoradiography. Nucleotide sequences of synthetic RNAs are listed in Additional File 6.

To analyze pre-miRNAs and their products in reconstitution assays, 5'- ^32^P- pre-miR-30a (20 nM) or 5'-^32^P- pre-miR-24-1, was incubated with 20 ng GST-Ago2, GST-TRBP and/or Dicer at 37°C for 30 min before addition of cold RNA targets for 60 min. 50 percent of the reaction volume was analyzed on 15% UREA PAGE. The remaining 50 percent was mixed with native RNA loading buffer (6% w/v Ficoll 400, 0.08% bromophenol blue (w/v) and 4 mM Tris pH7.5) and analyzed on 0.75 mm 15% native polyacrylamide (19:1), 0.5X TBE gel at 125V (80-25 mA). Radiolabeled RNA products were detected by autoradiography.

### *Dicer-null *mouse embryonic fibroblasts (MEFs)

The inducible *Dicer-null *MEFs were immortalized, cultured and induced as previously described [31].

### Immunoprecipitations and Northern blot analyses

*Dicer-null *MEF lysates were incubated with 20 μL of protein G agarose beads (Roche #11 243 233 001) pre-bound to 2 μL of non-immune mouse serum or 20 μL of anti-Ago 2A8 antibody [33] at 4°C overnight. Agarose beads were then washed five times in lysis buffer (20 mM Tris pH7.5, 200 mM NaCl, 2.5 mM MgCl_2 _and 0.5% Triton X-100) containing protease inhibitors (BMB #11697498001). 10 percent of total protein G agarose volume was used for Western blot and 30 percent for cleavage assay.

Northern blot analyses of total RNA isolated from *Dicer-null *MEFs (miRVana, Ambion) were performed as previously described [43,44]. 5'-^32^P- let-7a or let-7a* DNA antisense probes were used to detect let-7a and let-7a* expression. For loading control, miRNA expression was normalized against the expression of U6, detected with a DNA antisense probe.

## Authors' contributions

GST participated in the design of the study, carried out the *in vitro *assays and co-drafted the manuscript. BGG and DM carried out the purification of recombinant proteins. XL assisted GST in experimental work with *Dicer-null *cells. MK co-designed and supervised the study, compiled the figures and co-drafted the manuscript. All authors read and approved the final manuscript. The authors declare that they have no competing interests.

## Supplementary Material

Additional File 1 **In vitro reconstitution of miR-30 miRNP**. Recombinant Ago2 and Dicer Pare sufficient to recapitulate processing of 5'P-pre-miR-30a and loading of miR-30a* into Ago2 *in vitro*. Click here for file

Additional File 2 **In vitro RISC activity of pre-miR-103-2 miRNP**. Pre-miR-103-2 directs 5' -and 3' - arm target cleavage. Click here for file

Additional File 3 **Unstructured, single stranded RNA and targets**. Sequence of 73nt single stranded RNA aligned to the sequences of the 5' - and 3' -end targets. Click here for file

Additional File 4 **Dicer Western Blot**. Dicer detection in *Dicer+/- *and *Dicer-/- *MEFs. Click here for file

Additional File 5 **Purified recombinant Ago2, TRBP and Dicer**. Silver stain and Western Blot. Click here for file

Additional File 6 **Synthetic RNAs and DNA probes**. Sequences of synthetic oligonucleotides used in the studies. Click here for file
