# Supplementary material for: Clarifying mammalian RISC assembly in vitro
Source: BMC Mol Biol. 2011 Apr 29;12:19. doi: 10.1186/1471-2199-12-19 (PMC3112105; doi:10.1186/1471-2199-12-19)
Supplement: Additional File 1 — In vitro reconstitution of miR-30 miRNP. Recombinant Ago2 and Dicer Pare sufficient to recapitulate processing of 5'P-pre-miR-30a and loading of miR-30a* into Ago2 in vitro. [file 1471-2199-12-19-S1.PDF]

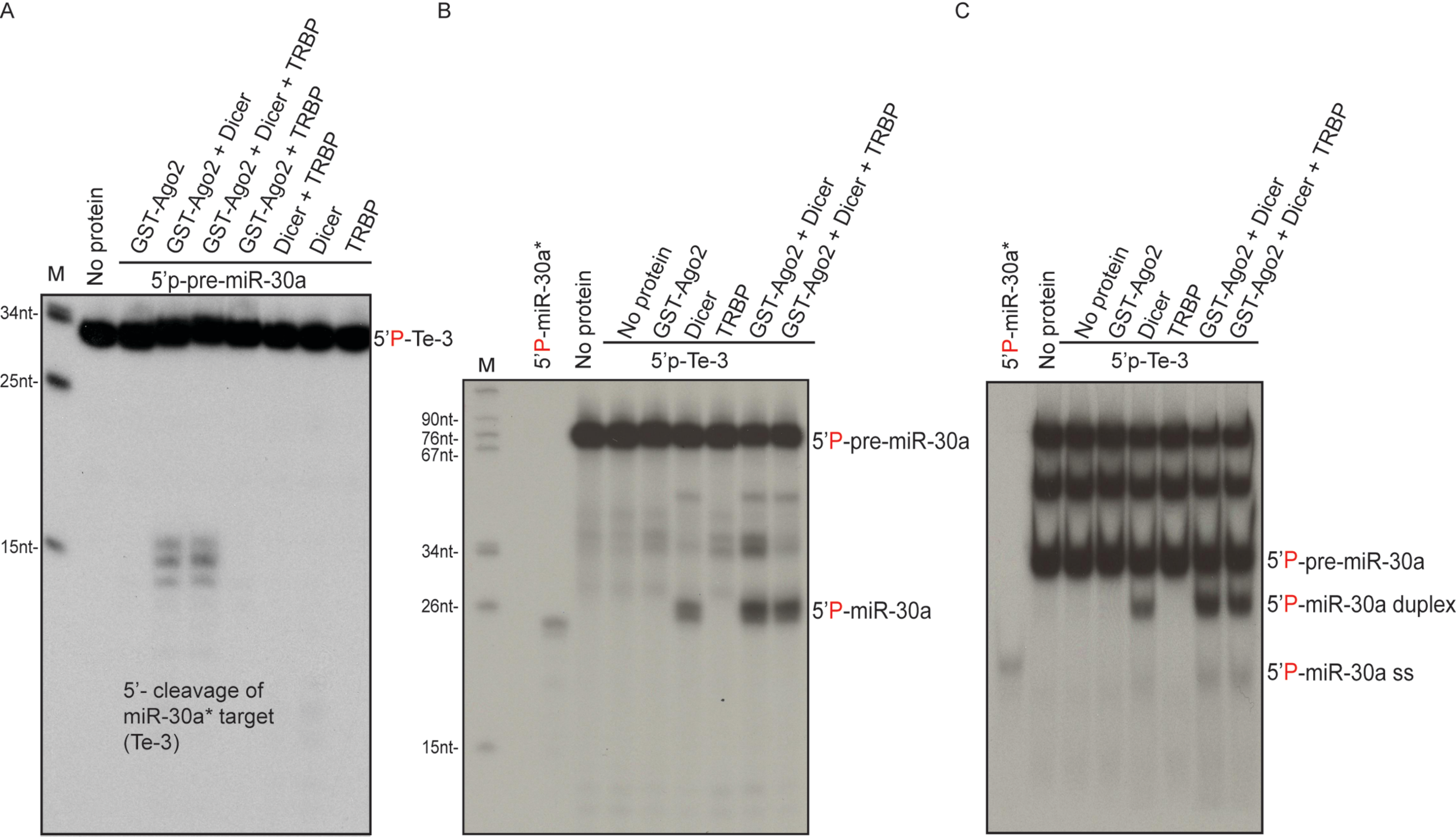

A
M
No protein
GST-Ago2
GST-Ago2 + Dicer
GST-Ago2 + Dicer + TRBP
GST-Ago2 + TRBP
Dicer + TRBP
Dicer
TRBP
5'p-pre-miR-30a
34nt-
25nt-
15nt-
5'P-Te-3
5'- cleavage of miR-30a* target (Te-3)
B
M
5'P-miR-30a*
No protein
No protein
GST-Ago2
Dicer
TRBP
GST-Ago2 + Dicer
GST-Ago2 + Dicer + TRBP
5'p-Te-3
90nt-
76nt-
67nt-
34nt-
26nt-
15nt-
5'P-pre-miR-30a
5'P-miR-30a
C
5'P-miR-30a*
No protein
No protein
GST-Ago2
Dicer
TRBP
GST-Ago2 + Dicer
GST-Ago2 + Dicer + TRBP
5'p-Te-3
5'P-pre-miR-30a
5'P-miR-30a duplex
5'P-miR-30a ss

Recombinant Ago2 and Dicer are sufficient to recapitulate processing of 5′P-pre-miR-30a and loading of miR-30a* into Ago2 *in vitro*. A. Recombinant Ago2, Dicer and TRBP were pre-incubated with 5′-P-pre-miR-30a prior to the addition of radiolabeled 3′-arm target (Te-3). Predicted size of 5′-cleavage product of Te-3 is 15 nt. Additional cleavage products indicate different sizes of miR-30a* generated by Dicer. **B.** Experiments were performed as in (A), except with radiolabeled pre-miR-30a and unlabeled 3′ arm target (Te-3). 50% of the total volume of each reaction was analyzed on 15% Urea PAGE and detected by autoradiography. **C.** The remaining 50% of each reaction was analyzed by native-PAGE. 5′-P^{32}-miR-30a * was used as size marker for single-stranded miRNA. Upper bands likely represent different conformations of pre-miR-30a.
