# Supplementary material for: Clarifying mammalian RISC assembly in vitro
Source: BMC Mol Biol. 2011 Apr 29;12:19. doi: 10.1186/1471-2199-12-19 (PMC3112105; doi:10.1186/1471-2199-12-19)
Supplement: Additional File 2 — In vitro RISC activity of pre-miR-103-2 miRNP. Pre-miR-103-2 directs 5' -and 3' - arm target cleavage. [file 1471-2199-12-19-S2.PDF]

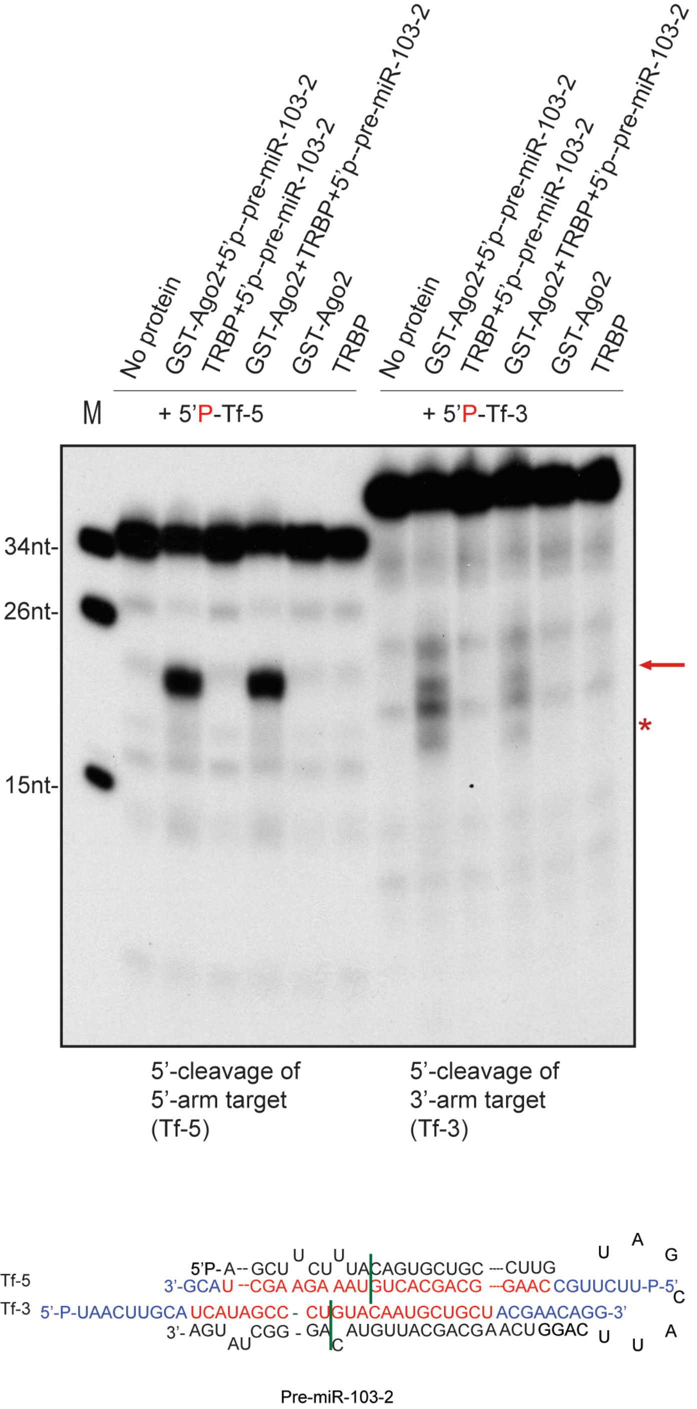

No protein
GST-Ago2+5'p-pre-miR-103-2
TRBP+5'p-pre-miR-103-2
GST-Ago2+TRBP+5'p-pre-miR-103-2
GST-Ago2
TRBP
No protein
GST-Ago2+5'p-pre-miR-103-2
TRBP+5'p-pre-miR-103-2
GST-Ago2+TRBP+5'p-pre-miR-103-2
GST-Ago2
TRBP
M
+ 5'P-Tf-5
+ 5'P-Tf-3
34nt-
26nt-
15nt-
*
5'-cleavage of 5'-arm target (Tf-5)
5'-cleavage of 3'-arm target (Tf-3)
5'P-A--GCU U CU U UACAGUGCUGC ---CUUG U A G
Tf-5 3'-GCAU --CGA AGA AAUGUCACGACG ---GAAC CGUUCUU-P-5' C
Tf-3 5'-P-UAACUUGCA UCAUAGCC - CUGUACAAUGCUGCU ACGAACAGG-3'
3'- AGU AU CGG - GA C AUGUUACGACGA ACU GGAC U U A
Pre-miR-103-2

Pre-miR-103-2 directs 5 ′-and 3′- arm target cleavage. Recombinant Ago2 and TRBP were pre-incubated with gel-purified 5′-P-pre-miR-103-2. Radiolabeled gel-purified targets complementary to the 5′-arm (Tf-5) or 3′-arm (Tf-3) of 5′P- pre-miR-103-2 were then added. Predicted size of 5′- cleavage product of 5′- and 3′-arm target is 20 nt and 19 nt respectively. Asterisks indicate additional 5′- cleavage products of 3′-arm targets
