# Supplementary figures and images for: Clarifying mammalian RISC assembly in vitro
Source: BMC Mol Biol. 2011 Apr 29;12:19. doi: 10.1186/1471-2199-12-19 (PMC3112105; doi:10.1186/1471-2199-12-19)

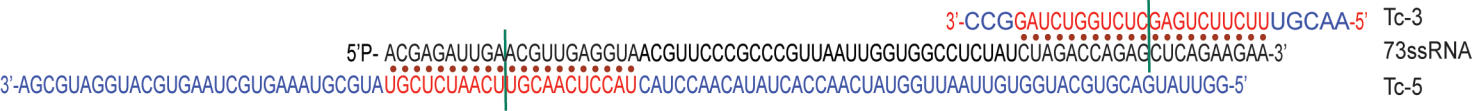

Supplement: Additional File 3 — Unstructured, single stranded RNA and targets. Sequence of 73nt single stranded RNA aligned to the sequences of the 5' - and 3' -end targets. [file 1471-2199-12-19-S3.PDF]

WB

---

Dicer<sup>+/-</sup>  
Dicer<sup>-/-</sup>

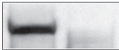

– Dicer

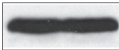

– Tubulin

Supplement: Additional File 4 — Dicer Western Blot. Dicer detection in Dicer+/- and Dicer-/- MEFs. [file 1471-2199-12-19-S4.PDF]
