# Supplementary material for: Clarifying mammalian RISC assembly in vitro
Source: BMC Mol Biol. 2011 Apr 29;12:19. doi: 10.1186/1471-2199-12-19 (PMC3112105; doi:10.1186/1471-2199-12-19)
Supplement: Additional File 5 — Purified recombinant Ago2, TRBP and Dicer. Silver stain and Western Blot. [file 1471-2199-12-19-S5.PDF]

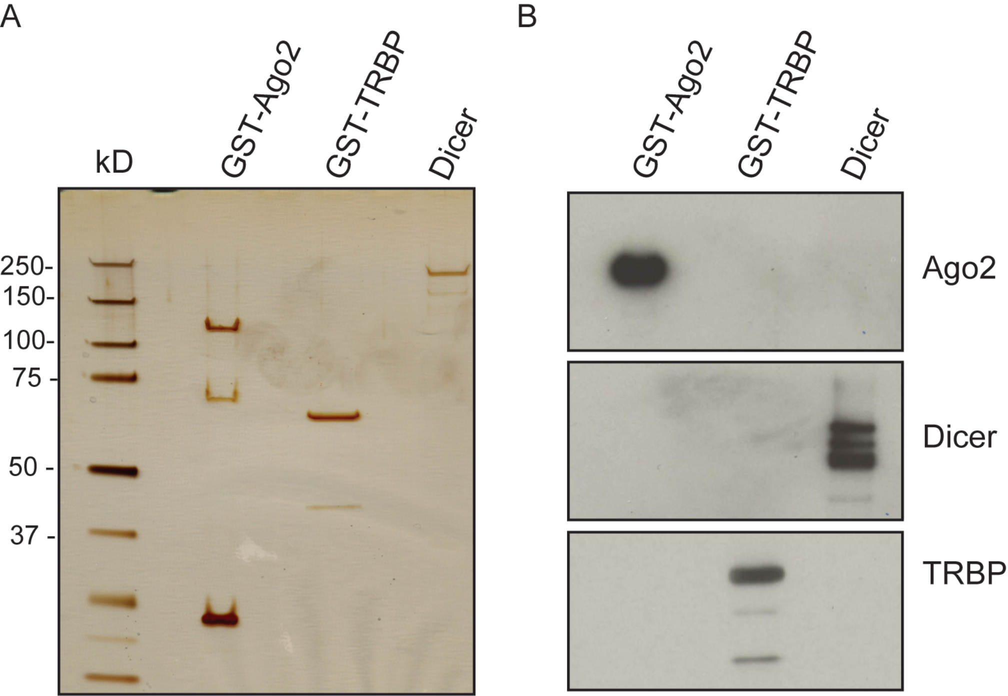

A
kD
GST-Ago2
GST-TRBP
Dicer
250-
150-
100-
75 -
50 -
37 -
B
GST-Ago2
GST-TRBP
Dicer
Ago2
Dicer
TRBP

Purified recombinant Ago2, TRBP and Dicer. A. Silver stained NuPAGE of 100ng of recombinant GST-Ago2, GST-TRBP and Dicer. **B.** Western blot analyses were performed on 100 ng of purified recombinant GST-Ago2, GST-TRBP and Dicer after elution with glutathione and anion exchange chromatography (GST-Ago2 and GST-TRBP) with antibodies as indicated.
