# Supplementary material for: Clarifying mammalian RISC assembly in vitro
Source: BMC Mol Biol. 2011 Apr 29;12:19. doi: 10.1186/1471-2199-12-19 (PMC3112105; doi:10.1186/1471-2199-12-19)
Supplement: Additional File 6 — Synthetic RNAs and DNA probes. Sequences of synthetic oligonucleotides used in the studies. [file 1471-2199-12-19-S6.PDF]

Synthetic RNAs

73 nt ss RNA

5′p - ACGAGAUUGAACGUUGAGGUAACGUUCCCGCCCGUUAAUUGGUGGCCUC
UAUCUAGACCAGAGCUCAGAAGAA - 3′

Pre-miR-24-1

5′p - UGCCUACUGAGCUGAUAUCAGUUCUCAUUUUACACACUGGCUCAGUU
CAGCAGGAACAG - 3′

Pre-miR-30a

5′p - UGUAAACAUCCUCGACUGGAAGCUGUGAAGCCACAGAUGGGCUUUCA
GUCG GAUGUUUGCAGC - 3′

Pre-miR-103-2

5′p - AGCUUCUUUACAGUGCUGCCUUGUAGCAUUCAGGUCAAGCAGCAUUG
UACAGGGCUAUGA - 3′

Pre-miR-138-2

5′p - AGCUGGUGUUGUGAAUCAGGCCGACGAGCAGCGCAUCCUCUUACCC
GGCUA UUUCACGACACCAGGGUU - 3′

Ta-5

5′ - GUCACACUGAUAUCAGCUCAGUAGGCAGGC - 3′

Ta-3

5′ - GCAACUGUUCCUGCUGAACUGAGCCAAGAC - 3′

Tb-5

5′ - GAUGCGGCCUGAUUCACAACACCAGCUUAC - 3′

Tb-3

5′ - GUCCAAAACCCUGGUGUCGUGAAAUAGCAA - 3′

Tc-5

5′p - GGUUAUGACGUGCAUGGUGUUAAUUGGUAUCAACCACUAUACAACCUAC

UACCUCAACGUUCAAUCUCGUAUGCGUAAAGUGCUAAGUGCAUGGAUGCGA-3′

Tc-3

5′ - AACGUUUCUUCUGAGCUCUGGUCUAGGCC - 3′

Td-5

5′ - UAUACAACCUACUACCUCAUC - 3′

Td-3

5′ - UUCAUUGAAAGACAGUAGAUUGUAUAGCCG - 3′

Te-5

5′ - ACAAGCUUCCAGUCGAGGAUGUUUACACCA - 3′

Te-3

5′ - AACGCUGCAAACAUCCGACUGAAAGAAACG - 3′

Tf-5

5′ - UUCUUGCCAAGGCAGCACUGUAAAGAAGCUACG - 3′

Tf-3

5′ - UAACUUGCAUCAUAGCCCUGUACAAUGCUGCUACGAACAGG - 3′

DNA probes

Let7a Guide DNA Anti-sense (A/S)

5′ - AACTATACAACCTACTACCTCA - 3′

*Let7a * DNA A/S*

5′ - GAAAGACAGTAGATTGTATAG - 3′

U6 DNA A/S

5′ - GTATATGTGCTGCCGAAGCGAGCAC - 3′
